# Circadian Rhythms of Retinomotor Movement in a Marine Megapredator, the Atlantic Tarpon, *Megalops atlanticus*

**DOI:** 10.3390/ijms18102068

**Published:** 2017-09-28

**Authors:** Kristin L. Kopperud, Michael S. Grace

**Affiliations:** College of Science, Florida Institute of Technology, 150 West University Blvd, Melbourne, FL 32901, USA; kkopperud2010@my.fit.edu

**Keywords:** retinomotor movement, photoreceptors, circadian rhythm

## Abstract

Many ecologically and economically important marine fish species worldwide spend portions of their lives in coastal regions that are increasingly inundated by artificial light at night. However, while extensive research illustrates the harmful effects of inappropriate light exposure on biological timing in humans, rodents and birds, comparable studies on marine fish are virtually nonexistent. This study aimed to assess the effects of light on biological clock function in the marine fish retina using the Atlantic tarpon (*Megalops atlanticus*) as a model. Using anti-opsin immunofluorescence, we observed robust rhythms of photoreceptor outer segment position (retinomotor movement) over the course of the daily light–dark cycle: cone outer segments were contracted toward the inner retina and rods were elongated during the day; the opposite occurred at night. Phase shifting the daily light–dark cycle caused a corresponding shift of retinomotor movement timing, and cone retinomotor movement persisted in constant darkness, indicating control by a circadian clock. Constant light abolished retinomotor movements of both photoreceptor types. Thus, abnormally-timed light exposure may disrupt normal *M. atlanticus* clock function and harm vision, which in turn may affect prey capture and predator avoidance. These results should help inform efforts to mitigate the effects of coastal light pollution on organisms in marine ecosystems.

## 1. Introduction

Predictable abiotic variations in the natural environment including the daily light–dark cycle and seasonal fluctuations of day length and temperature exert selective pressures that have shaped much of life on Earth, permitting organisms to exploit temporal as well as spatial niches [1,2,3]. Most organisms do not simply respond to cyclic changes in the environment, however. Rather they have evolved precise internal timekeeping mechanisms that allow anticipation of and preparation for environmental change, and coordination of physiological and behavioral states with environmental characteristics [1,2,3,4,5,6]. Importantly, rhythmic physiological and behavioral outputs of biological clocks persist even when natural time cues may be temporarily obscured [2,7].

Among vertebrate animals, biological clocks—particularly circadian (daily) clocks entrained by the daily light–dark cycle—have been most thoroughly studied in mammals [8,9], despite the fact that clocks are ubiquitous among vertebrate taxa. Coastal marine fish represent particularly fascinating animals in which to study clock function because they are subject to environmental cycles of multiple distinct periodicities (daily, tidal and lunar), and because many coastal marine fish have significant ecological and economic value. Even so, biological timekeeping mechanisms and the rhythms they control are poorly studied in fish outside the freshwater model organism *Danio rerio* [7,10], morphological examinations in a few other freshwater species including the green sunfish (*Lepomis cyanellus*) [11,12,13], bluegill (*Lepomis macrochirus*) [14] and cichlids [15,16,17], and limited studies of clock operation in some marine species such as the bluestriped grunt (*Haelmulon sciurus*) [18,19] and grey snapper (*Lutjanus griseus*) [19].

The daily light–dark cycle is the most important zeitgeber (time-keeper) for entrainment of circadian clocks to environmental time, and disruption of the normal light–dark cycle can have profoundly negative impacts on the health and well-being of eukaryotic organisms. Artificial light at night (LAN) arising from increasingly rapid coastal development may disrupt circadian clock function in coastal marine species [5,20], and interfere with organisms’ ability to respond in functionally appropriate ways to the natural daily light–dark cycle. Unfortunately, however, while extensive research has been conducted on the harmful effects of light at night on circadian function in humans, rodents and migratory birds [5,20,21], comparable studies on marine fish are lacking.

**Figure 1:**
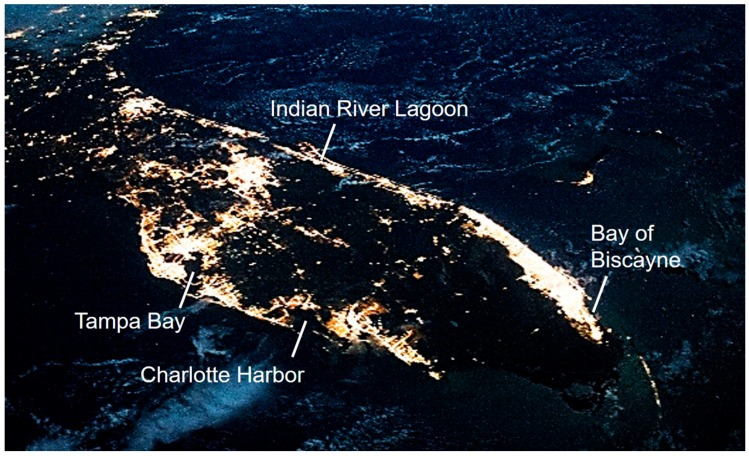
Florida coastline at night. Photograph from the International Space Station, October 2014, illustrating light pollution along the coasts of Florida, particularly surrounding some of the most important marine fish nursery habitats (labeled) in the state. Photo credit: ISS Crew Earth Observations Facility and Johnson Space Center.

Many ecologically and economically important marine species inhabit coastal estuarine ecosystems including those along much of the Florida coastline, many of which serve as critically important nursery habitats. However, it is precisely these coastal areas that have experienced the highest rates of development in recent decades [22]; development has brought habitat destruction [23], and increased the incidence and likely the magnitude of LAN (Figure 1).

The study presented here was designed to investigate circadian clock function in the Atlantic tarpon (*Megalops atlanticus* Valenciennes, 1847), a visually-guided predator [24,25] which spends much of its early life in coastal nursery habitats. Because the light-sensitive retina also contains a circadian clock in many vertebrate species (including *M. atlanticus*, which exhibits a circadian rhythm of visual sensitivity [26]), we set out to investigate the effects of light on clock function, here using retinomotor movement (RM) of photoreceptor outer segments as an assay. We assessed the extent of RM in a normal light–dark cycle, in phase-shifted light–dark cycles, in constant darkness, and in constant light. Our results show that the tarpon retina contains a circadian clock that drives physiologically-relevant, rhythmic cellular changes which can be phase-shifted and suppressed by alterations of the normal light–dark cycle. These results are discussed in the context of *M. atlanticus* ecology, coastal development, and mitigation of the potentially harmful effects of light at inappropriate times in coastal ecosystems.

## 2. Results

### 2.1. Tarpon Exposed to 12L:12D (LD)

**Figure 2:**
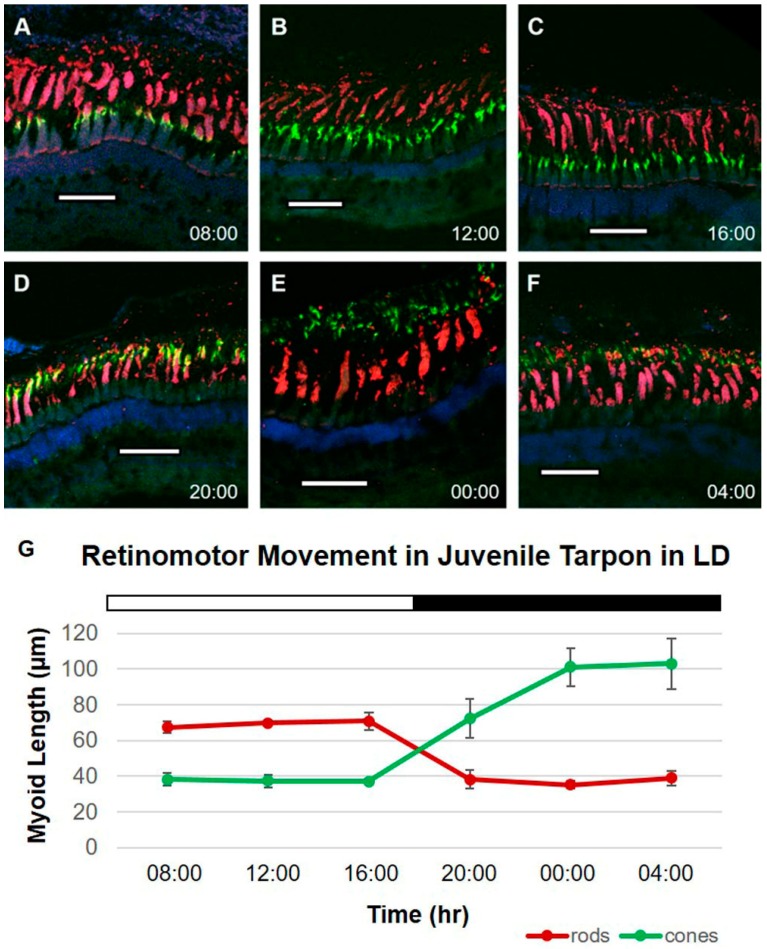
Immunolabeled retinal cross-sections and myoid lengths of juvenile *Megalops atlanticus* exposed to a 12L:12D lighting regime. Red: anti-rod opsin; green: anti-cone opsin; blue: DAPI. Scale bar: 100 µm. (**A**–**C**) Cone outer segments (COS) are retracted toward the outer limiting membrane (OLM) and rod outer segments (ROS) are extended toward the retinal pigmented epithelium (RPE) during light hours (lights on 07:00 to 19:00); (**D**) At 20:00, COS and ROS are in intermediate positions between OLM and RPE; (**E**,**F**) At 00:00 and 04:00, ROS are contracted toward the OLM and COS are extended toward the RPE; (**G**) Mean rod and cone myoid lengths over the 12:12 LD cycle (white bar = lights on; black bar = lights off). Error bars represent standard deviation; *n* = 3 animals per time point.

The retinas of juvenile tarpon (*N* = 18) were analyzed with immunofluorescence following a nine-day exposure (five-day entrainment period followed by a four-day treatment period) to a 12L:12D photocycle (LD). Thirty rod myoid and thirty cone myoid measurements were obtained from micrographs of each of six fish from three replicate populations sampled at 4 h time points across 24 h periods (Figure 2A–F). Means of each photoreceptor type were obtained across replicates for each time point (*t*_x_; Table 1) and were plotted over time (Figure 2G).

During light hours of the photocycle (*t*_8_, *t*_12_ and *t*_16_), rod myoid lengths ranged from 65.51 to 76.09 µm (Table 1), and were significantly longer (*p* < 0.001) than those sampled during dark hours (*t*_20_, *t*_0_ and *t*_4_), which ranged from 34.01 to 44.05 µm. Conversely, cone myoid lengths were significantly shorter during light hours (*p* < 0.001) than during dark hours. Cone myoid lengths ranged from 34.44 to 41.97 µm in light and 62.82 to 117.29 µm in darkness. Significant differences in myoid length between time points were determined with separate Tukey tests for rods (Table S2) and cones (Table S3).

### 2.2. Effects of Constant Darkness (DD)

**Figure 3:**
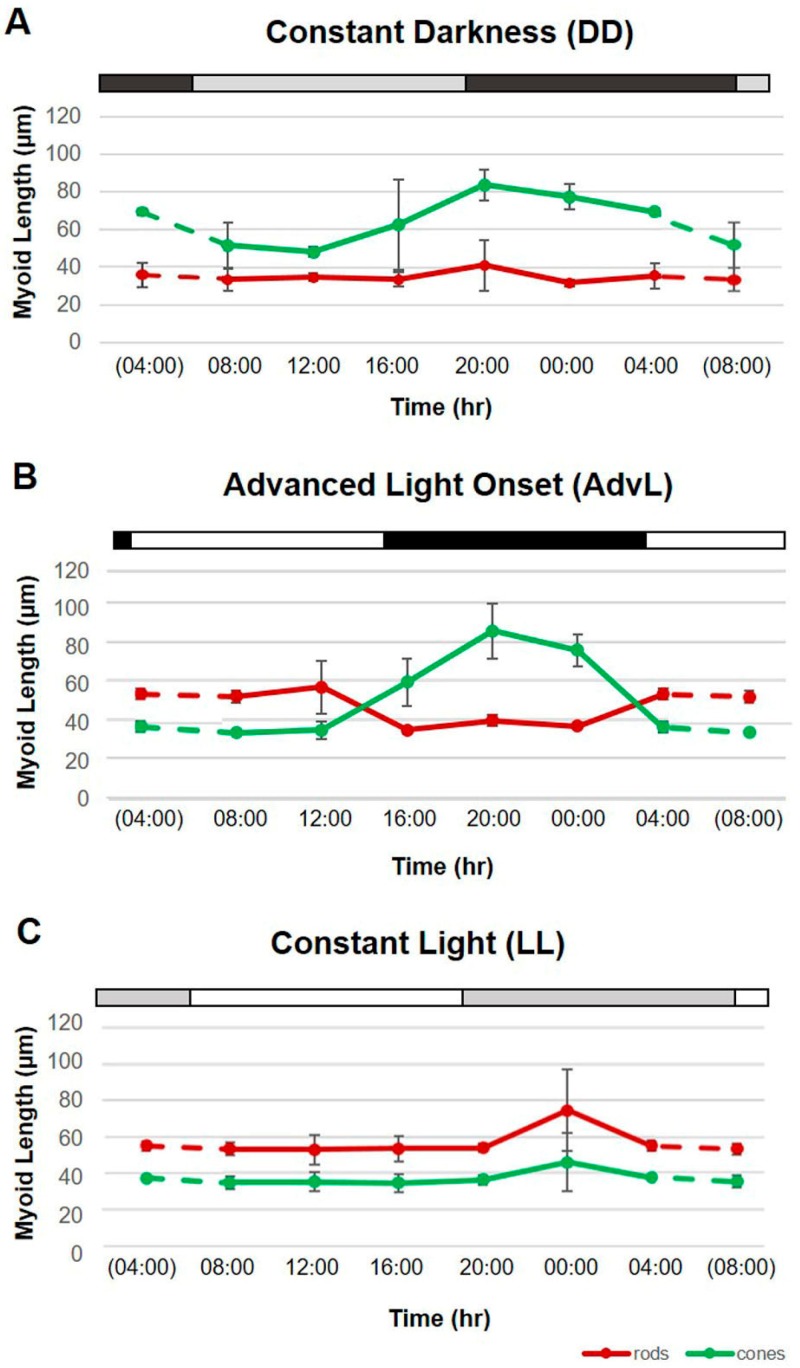
Mean rod and cone myoid lengths in juvenile *Megalops atlanticus* exposed to experimental lighting regimes. Tarpon (*n* = 6) were analyzed at 4 h intervals over a 24 h period (*x*-axis), three replicates per time point for each lighting regime. Myoid length (µm; *y*-axis) of rods (red line) and cones (green line). Dashed lines are extrapolations from collected data points to data from previous or successive time points within the same experimental group (parentheses). Error bars represent standard deviation. Shaded bar at the top of graph represents lighting condition at time of testing. (**A**) Constant darkness (DD). Dark gray bar = subjective night, light gray bar = subjective day; (**B**) Advanced (4 h) light onset (AdvL; lights-on at 03:00). Black bar = dark, white bar = light; (**C**) Constant light (LL). Gray bar = subjective night, white bar = subjective day.

To determine whether rhythms of rod and cone myoid length (i.e., retinomotor movement) are governed by a circadian clock, tarpon were exposed to constant darkness (DD; *N* = 18) for a four-day treatment period following an entrainment period under a 12L:12D photocycle. Rod and cone myoid lengths were obtained from micrographs from each specimen, the means of which were calculated across time points (Table 1) and were plotted over time (Figure 3A) as above.

A Kruskal–Wallis test between time points during subjective day (the hours during which fish would have experienced light) and those occurring during subjective night revealed that rod myoid lengths ranged from 27.58 to 56.59 µm but that length did not change significantly across time points (*p* = 0.727). However, a Welch’s *F*-test between subjective light and subjective dark hours indicated that significant differences existed among time points in cone myoid lengths (*p* = 0.002). As in LD, cone myoid length increased significantly during subjective night, ranging from 68.18 to 92.14 µm, and remained low during subjective day, ranging from 35.25 to 39.67 µm. Significant differences between subjective light and subjective dark hours were determined with a Games–Howell post-hoc test on cone myoid lengths (Table S4).

### 2.3. Effects of Phase-Advancing the LD Cycle (AdvL)

To determine whether rhythms of retinomotor movement in juvenile tarpon could entrain to a drastic and abrupt advance of the of the 12L:12D photocycle, lights were set to come on 4 h earlier during the treatment period (AdvL) than during the entrainment period. The retinas of juvenile tarpon (*N* = 18) were harvested at 4 h intervals over a 24 h period and were analyzed with immunohistochemistry as above. The means of thirty rod myoid and thirty cone myoid measurements from three specimens sampled during a given time point (*t*_x_) were obtained from micrographs of photoreceptor immunofluorescence and means of each photoreceptor type (Table 1) were plotted over time (Figure 3B) to determine whether rhythms of retinomotor movement adjusted to the advanced photocycle.

Rod myoid lengths ranged from 47.87 to 74.67 µm during light hours, and from 34.26 to 43.54 µm during dark hours in fish in AdvL. A Kruskal–Wallis test revealed that significant differences in rod myoid length existed across time points (*p* = 0.013), and a Kruskal–Wallis post-hoc pairwise test was conducted to identify which time points differed (Table S5). Likewise, a one-way ANOVA between light and dark hours indicated that significant differences existed in cone myoid lengths across time points (*p* < 0.001). Cone myoid length increased significantly during dark hours, ranging from 49.89 to 100.42 µm, and decreased during light hours, ranging from 32.83 and 41.74 µm. Significant differences in cone myoid lengths between light and dark hours were determined with a Tukey post-hoc test (Table S6).

### 2.4. Effects of Exposure to Constant Light (LL)

To determine whether rhythms of rod and cone retinomotor movement are disrupted by light at inappropriate times, tarpon were exposed to constant light (LL; *N* = 18) for four days following an entrainment period under a 12L:12D photocycle. Rod and cone myoid lengths were obtained from micrographs obtained from each specimen. The means were calculated across time points (Table 1) and were plotted over time (Figure 3C) as above.

A Kruskal–Wallis test comparing rod myoid lengths in fish in LL between subjective day and subjective night revealed, as in DD, that length did not change significantly across time points (*p* = 0.215). However, unlike in DD, cone myoid length also did not differ significantly between subjective light and subjective dark hours (*p* = 0.780, Welch’s *F*-test). Rod myoid length ranged from 44.85 to 61.49 µm during subjective day, and from 50.99 to 100.02 µm during subjective night. Cone myoid length ranged from 29.04 to 40.30 µm during subjective day, and from 33.54 to 64.35 µm during subjective night.

## 3. Discussion

The work reported here was undertaken to assess whether retinal photoreceptor cells exhibit circadian rhythms of retinomotor movement in a marine fish species associated with potentially light-polluted habitats. This investigation was conducted as part of a larger effort to understand how alterations of the natural light–dark cycle may affect the biology of marine species inhabiting coastal and other areas inundated by artificial light at night (see [26]). The results presented here show that both rod and cone myoids exhibited robust changes in length over the course of the daily light–dark cycle. Such retinomotor movement (RM) results in optimal positioning of rod and cone outer segments for effective light capture according to time of day (cone outer segments closer to the incoming light during the day and rod outer segments closer to incoming light at night; see Table 1).

In order to determine whether the repositioning of tarpon retinal photoreceptor outer segments in LD is controlled, at least in part, by an endogenous biological clock, we subjected populations of fish to experimental lighting regimes designed to assess whether cyclic RM entrains to a phase-shifted LD cycle, and whether a rhythm persists in the absence of LD time cues. After entrainment to a 12L:12D photocycle followed by four days in constant darkness (DD), RM of rods was suppressed or abolished (see Figure 3A). Rods remained in the contracted state, with all mean lengths over the course of the day remaining under 41 µm. However, in constant darkness (DD), cone outer segment position continued to oscillate rhythmically with a period of approximately 24 h, implicating the involvement of an internal timekeeping mechanism [27]. Because experiments were conducted only in whole animals, the anatomical location of the clock is unknown.

**Figure 4:**
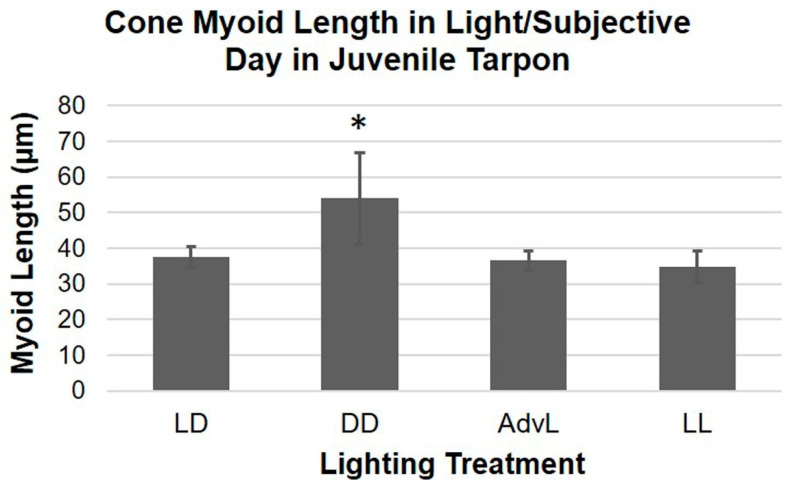
Comparison of cone retinomotor movement in light hours. Mean cone myoid lengths (µm) in light hours (LD and AdvL) or subjective day (DD and LL) were compared between lighting treatments. Error bars represent standard deviation. Significant difference (α = 0.05) denoted by (*).

Mean cone myoid length during subjective day in DD was significantly greater than during the light phase of LD, during subjective day in constant light, and during the light phase of a phase-shifted LD cycle (Figure 4). Results of a Kruskal–Wallis post-hoc pairwise test (Table S7; *p* < 0.001) indicated that cone OS did not contract in DD as fully as they did in the presence of light, possibly because of an additive effect of light and a biological clock through the interaction of dopamine and melatonin [27]. Light modulates photoreceptor outer segment position via dopamine D_2_ receptors [27], reducing adenylate cyclase activity, thus decreasing cyclic adenosine 3′,5′-monophosphate (cAMP) production [15]. Decreased levels of cAMP may induce cone contraction and inhibit melatonin synthesis. Darkness, in turn, inhibits dopamine synthesis in amacrine cells, and increases production of both melatonin and cAMP, resulting in microtubule-based cone elongation and melanin pigment granule aggregation in the RPE [27]. The results presented here are consistent with a model of light/dark and dopamine/melatonin interactions in the control of daily retinal changes in other vertebrate species [27,28,29].

After entrainment to a 12:12 LD cycle, additional populations of tarpon were exposed to a 4 h advance of the LD cycle (AdvL). After four days in this advanced photocycle, the rhythm of RM had become entrained to the new LD cycle, with rod and cone OS positions changing significantly between light and dark phases. Constant light (LL), however, suppressed RM of both rods and cones; neither photoreceptor type exhibited significant outer segment position change between subjective day and subjective night (see Figure 3B,C, respectively).

While circadian rhythms of retinomotor movement have been known for many years and have been studied in a small variety of vertebrate species (particularly a few model species and a very few wild vertebrates), the results described here extend our knowledge and the implications of these rhythms in important new ways. Perhaps most important, the effects of light on a circadian rhythm of photoreceptor cell biology described here indicate that exposure to artificial light at night may adversely affect normal cellular function, the process of vision, and perhaps other aspects of physiology and behavior in wild species living in light-polluted habitats.

Artificial light at night is increasingly recognized as a threat to living organisms in natural habitats, and human population growth has been occurring faster in coastal zones than other geographic areas [30], making these areas particularly vulnerable to light pollution. Marine turtles are well known to be adversely affected by inappropriate light at night [31,32,33], but they are not the only coastal organisms affected. For example, beach mice (*Peromyscus polionotus*) exhibited significantly abnormal food-gathering behavior when exposed to multiple types of artificial light in their otherwise natural habitat [34]. Invertebrates and aquatic animals are also affected. Moore et al. [35] reported that the normal diel vertical migration of *Daphnia* was significantly reduced under exposure to artificial light in a suburban lake, and the authors argued that there could be broad downstream ecological effects of this anomalous *Daphnia* behavior. Similar effects are seen in fish. Artificial light affects temporal migratory patterns [36] and depth selection [37] in Atlantic salmon (*Salmo salar*). The common observation that fish aggregate near artificial light sources (see [38]) is the basis for the development of light traps for collection of wild fish [39].

In fish and many other wild species, the potential health effects stemming from abnormal light exposure are very poorly known, if at all, but results from studies in humans suggest that broad health effects are likely. Abrupt shifts in the daily light–dark cycle (phase advance or delay) lead to desynchrony between internal clocks and environmental time, desynchrony in the mammalian suprachiasmatic nucleus [40] and the expression of jet lag. The effects can be severe: exposure to light during darkness can increase cancer risk and stimulate the progression of tumors and cancer [41,42,43,44,45] and can lead to dysregulation of the immune system [46]. Disruption of the circadian timing system can cause a variety of neurological, physiological and behavioral deficits including changes in brain microanatomy, obesity, and cognitive flexibility [47], and chronic desynchrony can increase mortality [48].

The results reported here show that constant light and advanced onset of light alter the circadian rhythm of retinomotor movement in *M. atlanticus*, and they provide a foundation for examining the broader effects of light on the molecular and cellular basis of circadian rhythms in marine vertebrate animals. It should be noted that the results reported here derive solely from analysis of juvenile *M. atlanticus*, but a circadian rhythm of retinomotor movement may exist from metamorphosis through adulthood. Elopomorph fish begin life as leptocephalus larvae inhabiting deep ocean waters, and they transform into juveniles as they migrate into inshore nursery habitats. The retinas of *M. atlanticus* leptocephali are highly rod-dominated and possibly lack cones altogether at early stages [49,50,51,52,53], and retinomotor movements appear to be absent in larvae and very early stage juveniles, but are robust in adults ([51]; S. Taylor and M. Grace, personal observation). Juveniles (and some adults occupying human-disturbed habitats) are most likely to have their circadian rhythms affected by light at inappropriate times because they inhabit protected inshore waters [24,25] that are most likely to be inundated by artificial light at night.

Photomechanical movements in the retina are thought to be adaptations to the photic environment [54], and control of retinomotor movements appears to be localized not just to the retina (they persist with severed optic nerve) but also within the retina (a spot of light focused on a portion of the retina leads to photomechanical adaptation of both photoreceptors and retinal pigmented epithelium) [15]. Altered environmental light may affect retina function specifically, perhaps directly interfering with optimal positioning of the photoreceptor complex for photon capture under given lighting conditions, but also interfering with circadian clock control of retinal light harvesting capability. Circadian clocks are known to be contained within the eye [55,56,57]; both retinal clocks and circadian oscillators located elsewhere in the body may be negatively affected by artificial light at night (see the discussion above regarding clock dysfunction and health effects).

Artificial light at night may cause problems beyond their effects on circadian clocks. For example, light history is well known to affect retinal development in a variety of marine and freshwater fish species. Abnormal light exposure can change the expression of opsin proteins in retinal photoreceptors [58,59,60,61,62], affect the spectral sensitivity of photoreceptor cells [61,62], and even change retinal architecture [63,64,65]. In most of these studies, abnormal light exposure occurred over the duration of development, but recently, we found that exposure to abnormal light spectra over very limited region of the developmental timecourse (2–4 months) can dramatically alter spectral sensitivity of the retina in *M. atlanticus* (L. Schweikert and M. Grace, in press).

Given the broad array of serious effects that abnormal timing of light exposure can cause in mammals and the increasingly abundant evidence for harmful effects of abnormal light exposure in fishes, it is reasonable to suspect that similarly serious effects may occur in all groups of non-mammalian vertebrates. A better understanding of how light affects photoreceptor and clock functions in marine fish will help define the effects of light pollution in the natural environment and will provide a basis for mitigating the likely harmful effects of light at inappropriate times that accompany the increasing coastal development along many of the world’s most productive marine habitats.

## 4. Methods

### 4.1. Animal Collection and Housing

Juvenile (“young-of-the-year”) *Megalops atlanticus* were captured via cast net in Brevard County drainage canals and in a mosquito control impoundment in Indian River County, Florida (*N* = 72). Animal collection and experimental procedures were conducted in accordance with Florida Fish and Wildlife Special Activity License SAL-15-1300-SRP (approved 7 November 2014) and Florida Institute of Technology Institutional Animal Care and Use Committee protocol 141013 (approved 11 November 2014).

Upon capture, juveniles were transferred in an insulated and aerated container filled with habitat water to the aquaculture facility at Florida Institute of Technology where they were held under a 12L:12D photocycle (12 h light, 12 h dark; lights on 07:00) using standard fluorescent room lighting. Fish were drip-acclimated from habitat water to laboratory water over a three-hour period or until salinity and temperature reached those of the quarantine system (24 ppt; 24 °C). Fish were then transferred to a quarantine tank (340 L) that contained extra aeration and mechanical filtration. Temperature was maintained between 24 and 25 °C and salinity between 24 and 25 ppt. Fish were treated with nitrofurazone antibiotic (7.6 mg·gal^−1^; [66]) for five days, after which 50% of the tank water was changed daily for five days to remove the antibiotic, and after which filtration was both mechanical and charcoal-based. To replace the natural slime coat that may have been lost during capture and transport, as well as to remove toxic ammonia, nitrite, nitrate and chlorine, twice the recommended dosage of Prime water conditioner (2 mL·gal^−1^; Seachem Laboratories, Madison, GA, USA) was added to tank water every day during the quarantine period.

Salinity and temperature were monitored daily, and ammonia, nitrite, nitrate and pH levels were tested every other day with an API Saltwater Master Test Kit (Mars Fishcare Inc., Chalfont, PA, USA). When parameters exceeded maximal acceptable ranges (0.5 ppm for ammonia and nitrite, and 40 ppm for nitrate) or pH was outside of the 7.8–8.1 range, 25% of the tank water was drained and replaced with a fresh mixture. Weekly water changes were performed for maintenance. Tarpon were fed a mixture of frozen krill and live mosquitofish (*Gambusia affinis*) every other day, starting approximately 5 days post-capture (i.e., after the antibiotic treatment ceased). Fish that were not immediately used for experimentation were transferred to holding tanks until needed.

### 4.2. Experimental Treatment

**Figure 5:**
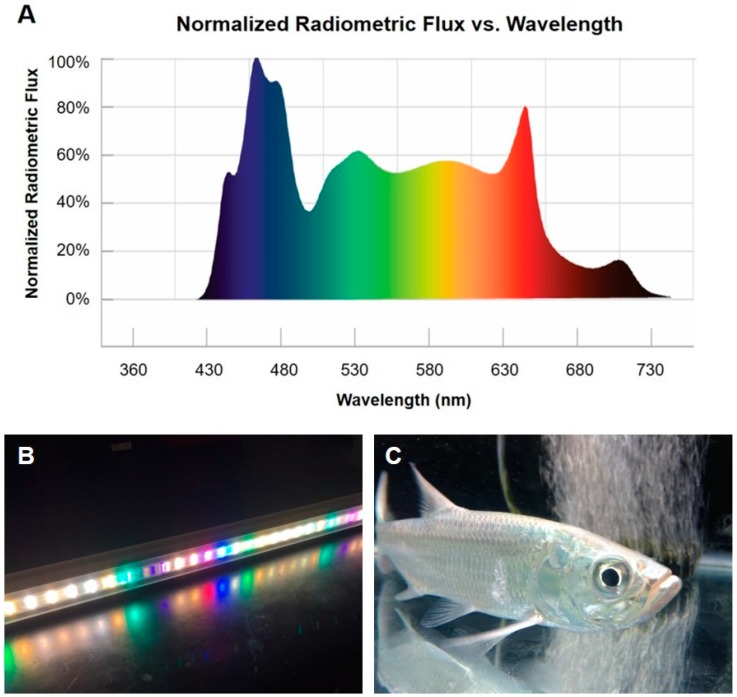
Custom LED light bar designed to replicate natural sunlight in experimental conditions: (**A**) normalized radiometric flux per unit of wavelength across the electromagnetic spectrum for the 5700K SolarMax LED light bar; (**B**) used to light experimental tanks during entrainment and during the light phase of LD treatments; and (**C**) juvenile tarpon in experimental tank under LED light.

After at least a two-week quarantine period, populations of six fish were exposed to experimental lighting treatments. Fish were housed in 208 L experimental tanks equipped with hang-on power filters (QuietFlow 55/75, Aqueon Products, Franklin, WI, USA) and aerated with a large airstone. Walls of tanks were covered in lightproof black film, and fish were exposed to broad-spectrum LED light (SolarMax 5700K white light, BML Horticulture, Austin, TX, USA; Figure 5) at an intensity of 37.23 W·m^−2^. Habitat light intensity was measured on a sunny day at mid-depth in the water column to be 36.14 W·m^−2^; lights were placed 5 cm from the water’s surface in lab aquaria to provide approximately the same intensity at mid-tank depth. Light intensities were measured with a LI-COR light meter, model LI-250A (LI-COR Biosciences, Lincoln, NE, USA).

All experimental fish were exposed to a five-day entrainment period composed of a 12L:12D photocycle (lights-on at 07:00), followed by a four-day treatment period. Treatment consisted of exposing fish to one of four lighting regimes (Figure S1): continued cyclic light (12:12, LD); constant darkness (DD); constant light (LL); or 4 h advanced onset of the daily light stimulus (AdvL).

Three replicate groups consisting of 6 fish each were created for each lighting condition (for a total of 18 fish per lighting condition). For each replicate, single fish were euthanized by cervical dislocation at 4 h intervals over a 24 h period beginning at 08:00 in each of the lighting treatment periods (LD, DD, AdvL or LL). For time points in the light, euthanasia and collection of eyes were performed in normal fluorescent room lighting. For dark time points in LD and AdvL and for DD sampling, euthanasia and collection of eyes were performed under dim red light. Standard length (SL) measurements of fish were obtained (Table 2), eyes were harvested, and eyecups were prepared by removing the anterior segment (cornea and lens). Right and left eyes were fixed in separate, labeled vials containing 4% paraformaldehyde and 15% picric acid in 0.1 M sodium phosphate buffer, and were stored in the laboratory with gentle shaking.

### 4.3. Immunofluorescence

Rod and cone photoreceptor position in relation to the outer limiting membrane (OLM; see Graphical Abstract) were analyzed with immunofluorescence to determine the extent of retinomotor movement in juvenile tarpon exposed to each of the four lighting regimes. After a minimum of 48 h of fixation, eyecups were infiltrated with 25% sucrose solution in 0.1 M Tris buffer for at least 24 h, and then embedded in Tissue-Tek OCT compound (Thermo Fisher Scientific, Waltham, MA, USA). Frozen (−20 °C) 15-μm retinal cross-sections were made through the central retina in the dorso-ventral plane using a cryostat (model CM1850; Leica Biosystems, Buffalo Grove, IL, USA). This region allowed sections to be made along the long axis of the photoreceptors for accurate quantitation of photoreceptor myoid lengths. Every third section was thaw-mounted onto gelatin-coated slides and dried at room temperature overnight. Slides were flooded with 4% paraformaldehyde fixative for 1 h, followed by serial rinses (five at 10 min each) with Tris-buffered saline (TBS; 0.02 M Tris buffer, 0.9% NaCl, pH 7.4).

Rod and cone photoreceptors were double-immunolabeled with mouse anti-rhodopsin (MAB-5316, 1:500 dilution; EMD Millipore, Billerica, MA, USA) and rabbit anti-cone opsin (CERN-906, 1:4000 dilution; Donated by W.J. DeGrip, University of Nijmegen, Nijmegen, The Netherlands) primary antisera diluted in TBS containing 0.25% λ-carrageenan, 1% bovine serum albumin, and 0.3% TritonX-100 (CBT). Both antisera have been well characterized for rod and cone opsins including those of *M. atlanticus* [52,53,67]. Primary antisera were omitted from one slide per retina as a negative control (slide incubated in TBS with CBT only). Retinal cross-sections were infiltrated with the solution containing primary antisera (or vehicle only) overnight, followed by serial 10-min rinses with TBS. Fluorophore-conjugated secondary antisera (goat anti-mouse and goat anti-rabbit IgG conjugated to AlexaFluor 555 and AlexaFluor 488, respectively; Life Technologies, Grand Island, NY, USA) were diluted 1:500 in TBS containing CBT. Slides were infiltrated with secondary antisera for 1 h in darkness, followed by serial 10-min rinses with TBS. Slides were coverslipped with Slowfade Gold anti-fade reagent with DAPI nucleic acid label (Life Technologies/Thermo Fisher Scientific, Waltham, MA, USA) and the coverslip perimeter sealed. Immunolabeling was viewed and photographed using a Nikon Eclipse 90i upright laser-scanning confocal microscope running EZ-C1 software (Nikon Instruments, Melville, NY, USA) using a D-F-T filter cube and 404, 488 and 561 nm lasers. Ten micrographs per retina were acquired at 20× magnification, in addition to a single image of the negative control.

### 4.4. Data Analysis

#### 4.4.1. Extent of Retinomotor Movement

Examination of photoreceptor outer segment position with respect to the outer limiting membrane (OLM) served as a metric for rod and cone retinomotor movement (see [68]). The extent of RMM was defined as the distance from the proximal edge of the rod or cone OS to the OLM. The extent of RMM was calculated for randomly-selected rods and cones (*n* = 30 per photoreceptor type; [19]) for three regions in each of ten micrographs per retina, utilizing NIS Elements AR software. The means of the three replicates for each time point were used to generate curves of the mean extent of RMM relative to time for rods and for cones in each lighting treatment (see sample data set in Table S1).

#### 4.4.2. Statistical Analyses

Mean rod myoid lengths and mean cone myoid lengths (µm) were examined separately using one-way ANOVAs to determine whether a significant difference (α < 0.05) existed across time points within each experimental treatment (LD, DD, AdvL and LL). Tests were run using SPSS software (IBM Corporation). Prior to running the tests, normal distribution of the residuals and homogeneity of variances were verified with Shapiro–Wilk and Levene’s tests, respectively. Welch’s F-tests of equality of means were run in cases where the homogeneity of variances assumption was violated but residuals were normally distributed. Kruskal–Wallis *H*-tests were used when residuals were not normally distributed and variances were not equal.

The null hypotheses stated that there was no significant difference between two means (or medians for non-parametric tests). A statistically significant result of *p* < 0.05 indicated rejection of the null hypotheses and acceptance of the alternative hypotheses that the mean myoid lengths were different. To determine which time point means significantly differed, a post-hoc Tukey test, Games–Howell test (when homogeneity of variances assumption was violated) or Kruskal–Wallis post-hoc pairwise test (when both homogeneity of variances and normality assumptions were violated) was performed.

## Figures and Tables

**Table 1:** Mean rod and cone myoid lengths (µm) for experimental analysis of retinomotor movement in juvenile *Megalops atlanticus* exposed to different lighting regimes. Thirty rod and thirty cone myoid lengths were obtained per specimen, three replicate groups per time point. Means (bold) and standard deviations (italics) calculated at each time point for rod and for cone myoids: (**A**) 12L:12D photocycle (LD); (**B**) Constant darkness (DD); (**C**) Advanced (4 h) light onset (AdvL; lights-on at 03:00); and (**D**) Constant light (LL). SD = standard deviation.

	**Mean Rod Myoid Lengths (µm)**							
	**LD**		**DD**		**AdvL**		**LL**	
**Time Point**	**Mean**	**SD**	**Mean**	**SD**	**Mean**	**SD**	**Mean**	**SD**
08:00	**67.42**	*3.14*	**33.33**	*6.02*	**54.05**	*3.40*	**53.29**	*3.27*
12:00	**69.90**	*1.31*	**34.73**	*2.04*	**59.05**	*13.94*	**52.73**	*8.35*
16:00	**70.77**	*4.86*	**33.56**	*3.81*	**36.47**	*1.91*	**53.59**	*6.98*
20:00	**38.24**	*5.06*	**40.98**	*13.59*	**41.51**	*2.71*	**53.72**	*2.39*
00:00	**35.19**	*2.18*	**31.58**	*1.95*	**38.48**	*0.61*	**74.50**	*22.14*
04:00	**38.93**	*4.13*	**35.36**	*6.57*	**55.38**	*2.92*	**55.05**	*2.62*
	**Mean Cone Myoid Lengths (µm)**							
	**LD**		**DD**		**AdvL**		**LL**	
**Time Point**	**Mean**	**SD**	**Mean**	**SD**	**Mean**	**SD**	**Mean**	**SD**
08:00	**38.25**	*3.59*	**51.42**	*11.95*	**35.14**	*0.88*	**34.80**	*3.35*
12:00	**37.27**	*3.52*	**48.05**	*2.72*	**36.45**	*4.68*	**35.25**	*5.36*
16:00	**37.17**	*1.43*	**62.54**	*23.77*	**61.58**	*12.21*	**34.53**	*4.85*
20:00	**72.21**	*10.92*	**83.56**	*8.17*	**88.44**	*14.57*	**36.32**	*2.66*
00:00	**101.08**	*10.56*	**77.28**	*6.85*	**78.47**	*8.37*	**46.01**	*15.92*
04:00	**103.14**	*14.14*	**69.33**	*1.71*	**37.98**	*3.00*	**37.45**	*1.51*

**Table 2:** Standard lengths (mm; rostral tip to distal caudal peduncle) of juvenile *Megalops atlanticus* included in experimental analysis of retinomotor movement. For each lighting treatment, six specimens were sampled every 4 h (time point) over 24 h, three replicates per time point. Specimens were exposed to one of four different lighting regimes: 12L:12D photocycle (LD); constant darkness (DD); advanced (4 h) light onset (AdvL; lights-on at 03:00); or constant light (LL).

**LD**	**Replicate**			**AdvL**	**Replicate**		
**Time Point**	**1**	**2**	**3**	**Time Point**	**1**	**2**	**3**
08:00	121	151	107	08:00	98	90	96
12:00	129	130	116	12:00	94	125	99
16:00	146	125	97	16:00	115	117	125
20:00	152	142	111	20:00	126	126	99
00:00	127	133	98	00:00	89	113	112
04:00	151	136	102	04:00	102	113	126
**DD**	**Replicate**			**LL**	**Replicate**		
**Time Point**	**1**	**2**	**3**	**Time Point**	**1**	**2**	**3**
08:00	102	92	84	08:00	118	91	92
12:00	116	98	88	12:00	96	83	108
16:00	117	89	87	16:00	101	100	85
20:00	98	104	86	20:00	101	93	88
00:00	105	105	99	00:00	120	100	101
04:00	110	98	99	04:00	109	94	86

## Supplementary Materials

Supplementary materials can be found at www.mdpi.com/1422-0067/18/10/2068/s1
