# Supplementary material for: Circadian Rhythms of Retinomotor Movement in a Marine Megapredator, the Atlantic Tarpon, Megalops atlanticus
Source: Int J Mol Sci. 2017 Sep 28;18(10):2068. doi: 10.3390/ijms18102068 (PMC5666750; doi:10.3390/ijms18102068)
Supplement: Supplementary file 1 [file ijms-18-02068-s001.pdf]

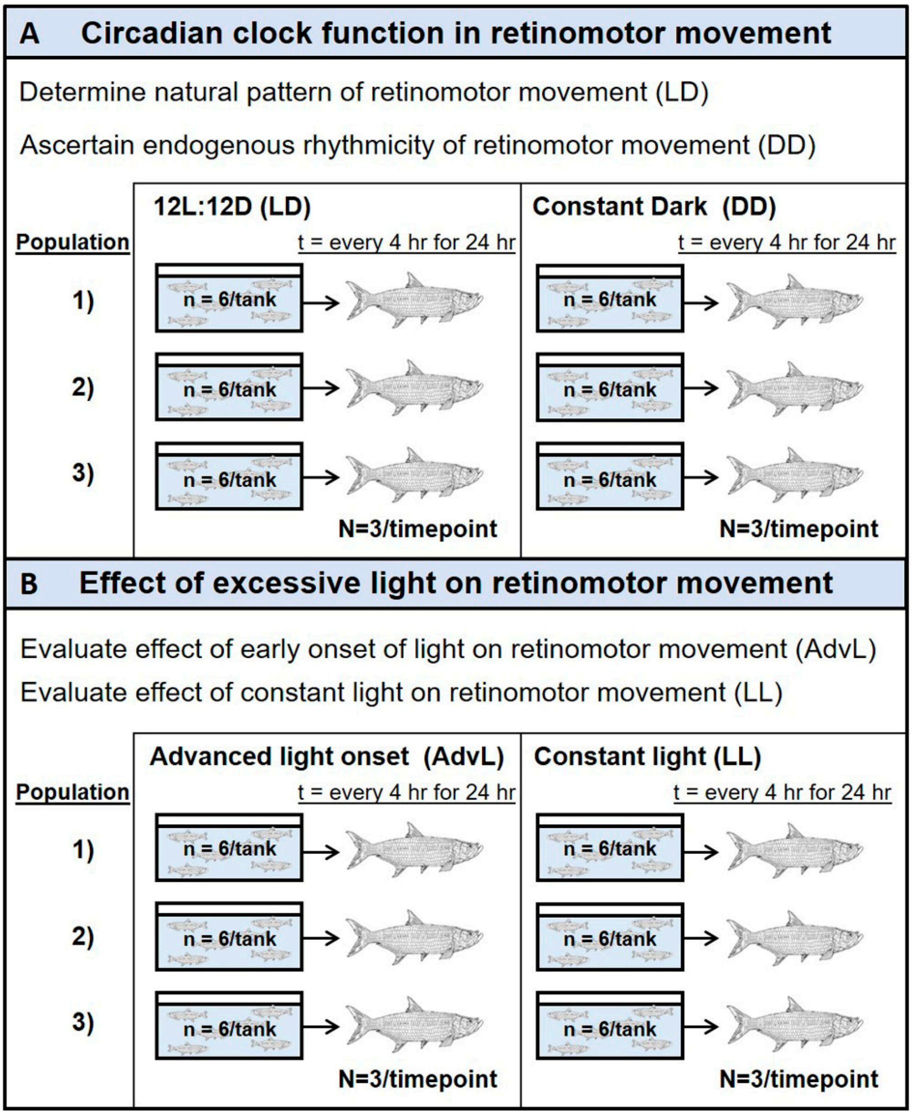

Figure S1. Objectives and flow chart for experiments investigating retinomotor movement in juvenile *Megalops atlanticus*. Four lighting "treatments" were designed to determine how oscillations of retinomotor movement changed over the course of the day (LD), and whether these changes continued in constant darkness (DD). Effects of unnatural light exposure were examined in fish exposed to an advanced onset of light exposure (AdvL; 4 hr advance) and constant light (LL). Three replicates were tested per lighting treatment.

Table S1. Sample data set of rod and cone myoid lengths (μm) for a single specimen used in studies of retinomotor movement. Distances of outer segments from the outer limiting membrane were obtained from thirty rods and cones per specimen.

**Dark-Adapted Rods**		**Dark-Adapted Cones**	
Item	Length (μm)	Item	Length (μm)
1	33.86	1	45.04
2	40.85	2	45.2
3	41.71	3	46.69
4	44.39	4	49.59
5	46.59	5	51.2
6	49.05	6	52.02
7	49.81	7	52.95
8	50.13	8	57.75
9	50.76	9	59.31
10	51.38	10	59.83
11	51.39	11	61.99
12	51.5	12	62.23
13	52.03	13	62.49
14	53.26	14	62.5
15	53.42	15	63.78
16	53.82	16	63.79
17	54.66	17	67.32
18	55.03	18	67.66
19	55.38	19	67.95
20	55.78	20	70.18
21	57.88	21	70.57
22	58.46	22	71.61
23	60	23	72.98
24	61.06	24	74.5
25	63.15	25	76.64
26	63.6	26	78.11
27	63.8	27	80.18
28	64.73	28	83.52
29	71.26	29	111.84
30	88.47	30	117.63
**Mean**	**54.91**	**Mean**	**66.9**
St.Dev	9.91	St.Dev	16.35

Table S2. Summary of Tukey post-hoc test on rod myoid length (µm) across time points in juvenile tarpon in LD. Box shading of Time point (i) indicates lighting condition at time of testing (white=light; black=dark). Statistically significant *p*-values ($\alpha \leq 0.05$) are in bold.

(i) Time point	(j) Time point	Mean Difference (i-j)	Standard Error	*p*-Value
**08:00**	12:00	-2.49	3.03	.958
	16:00	-3.36	3.03	.869
	20:00	29.18	3.03	**.000**
	00:00	32.23	3.03	**.000**
	04:00	28.49	3.03	**.000**
**12:00**	08:00	2.49	3.03	.958
	16:00	-.87	3.03	1.000
	20:00	31.66	3.03	**.000**
	00:00	34.72	3.03	**.000**
	04:00	30.98	3.03	**.000**
**16:00**	08:00	3.36	3.03	.869
	12:00	.87	3.03	1.000
	20:00	32.53	3.03	**.000**
	00:00	35.59	3.03	**.000**
	04:00	31.85	3.03	**.000**
**20:00**	08:00	-29.18	3.03	**.000**
	12:00	-31.66	3.03	**.000**
	16:00	-32.53	3.03	**.000**
	00:00	3.05	3.03	.907
	04:00	-.69	3.03	1.000
**00:00**	08:00	-32.23	3.03	**.000**
	12:00	-34.72	3.03	**.000**
	16:00	-35.59	3.03	**.000**
	20:00	-3.05	3.03	.907
	04:00	-3.74	3.03	.812
**04:00**	08:00	-28.49	3.03	**.000**
	12:00	-30.98	3.03	**.000**
	16:00	-31.85	3.03	**.000**
	20:00	.69	3.03	1.000
	00:00	3.74	3.03	.812

Table S3. Summary of Tukey post-hoc test on cone myoid length (μm) across time points in juvenile tarpon in LD. Box shading of Time point (i) indicates lighting condition at time of testing (white=light; black=dark). Statistically significant *p*-values ($\alpha \leq 0.05$) are in bold.

(i) Time point	(j) Time point	Mean Difference (i-j)	Standard Error	*p*-Value
**08:00**	12:00	.98	7.13	1.000
	16:00	1.08	7.13	1.000
	20:00	-33.96	7.13	**.005**
	00:00	-62.83	7.13	**.000**
	04:00	-64.89	7.13	**.000**
**12:00**	08:00	-.98	7.13	1.000
	16:00	.10	7.13	1.000
	20:00	-34.93	7.13	**.004**
	00:00	-63.80	7.13	**.000**
	04:00	-65.87	7.13	**.000**
**16:00**	08:00	-1.08	7.13	1.000
	12:00	-.10	7.13	1.000
	20:00	-35.03	7.13	**.004**
	00:00	-63.90	7.13	**.000**
	04:00	-65.97	7.13	**.000**
**20:00**	08:00	33.96	7.13	**.005**
	12:00	34.93	7.13	**.004**
	16:00	35.03	7.13	**.004**
	00:00	-28.87	7.13	**.016**
	04:00	-30.93	7.13	**.010**
**00:00**	08:00	62.83	7.13	**.000**
	12:00	63.80	7.13	**.000**
	16:00	63.90	7.13	**.000**
	20:00	28.87	7.13	**.016**
	04:00	-2.06	7.13	1.000
**04:00**	08:00	64.89	7.13	**.000**
	12:00	65.87	7.13	**.000**
	16:00	65.97	7.13	**.000**
	20:00	30.93	7.13	**.010**
	00:00	2.06	7.13	1.000

Table S4. Summary of Games-Howell post-hoc test on cone myoid length (μm) across time points in juvenile tarpon in DD. Box shading of Time point (i) indicates subjective lighting condition at time of testing (light grey=subjective day; dark grey=subjective night). Statistically significant *p*-values ($\alpha \leq 0.05$) are in bold.

(i) Time point	(j) Time point	Mean Difference (i-j)	Standard Error	*p*-Value
**08:00**	12:00	3.36	7.08	.993
	16:00	-11.12	15.36	.965
	20:00	-32.15	8.36	.113
	00:00	-25.86	7.95	.190
	04:00	-17.91	6.97	.378
**12:00**	08:00	-3.36	7.08	.993
	16:00	-14.49	13.81	.871
	20:00	-35.51	4.97	**.044**
	00:00	-29.23	4.25	**.041**
	04:00	-21.28	1.86	**.004**
**16:00**	08:00	11.12	15.36	.965
	12:00	14.49	13.81	.871
	20:00	-21.02	14.51	.716
	00:00	-14.74	14.28	.878
	04:00	-6.79	13.76	.991
20:00	08:00	32.15	8.36	.113
	12:00	35.51	4.97	**.044**
	16:00	21.02	14.51	.716
	00:00	6.28	6.15	.889
	04:00	14.23	4.82	.299
00:00	08:00	25.86	7.95	.190
	12:00	29.23	4.25	**.041**
	16:00	14.74	14.28	.878
	20:00	-6.28	6.15	.889
	04:00	7.95	4.07	.534
04:00	08:00	17.91	6.97	.378
	12:00	21.28	1.86	**.004**
	16:00	6.79	13.76	.991
	20:00	-14.23	4.82	.299
	00:00	-7.95	4.07	.534

Table S5. Summary of Kruskal-Wallis post-hoc pairwise test on rod myoid length (μm) across time points in juvenile tarpon in AdvL. Box shading of Time point (i) indicates lighting condition at time of testing (white=light; black=dark). Statistically significant *p*-values ($\alpha \leq 0.05$) are in bold.

Time point	Time point	Test Statistic	Standard Error	*p*-Value
**08:00**	12:00	-.67	4.36	.878
	16:00	11.33	4.36	**.009**
	20:00	6.00	4.36	.169
	00:00	7.67	4.36	.079
	04:00	-1.33	4.36	.760
**12:00**	08:00	-.67	4.36	.878
	16:00	12.00	4.36	**.006**
	20:00	6.67	4.36	.126
	00:00	8.33	4.36	.056
	04:00	-.67	4.36	.878
**16:00**	08:00	11.33	4.36	**.009**
	12:00	12.00	4.36	**.006**
	20:00	-5.33	4.36	.221
	00:00	-3.67	4.36	.400
	04:00	-12.67	4.36	**.004**
**20:00**	08:00	6.00	4.36	.169
	12:00	6.67	4.36	.126
	16:00	-5.33	4.36	.221
	00:00	1.67	4.36	.702
	04:00	-7.33	4.36	.092
**00:00**	08:00	7.67	4.36	.079
	12:00	8.33	4.36	.056
	16:00	-3.67	4.36	
	20:00	1.67	4.36	.702
	04:00	-9.00	4.36	**.039**
**04:00**	08:00	-1.33	4.36	.760
	12:00	-.67	4.36	.878
	16:00	-12.67	4.36	**.004**
	20:00	-7.33	4.36	.092
	00:00	-9.00	4.36	**.039**

Table S6. Summary of Tukey post-hoc test on cone myoid length (µm) across time points in juvenile tarpon in AdvL. Box shading of Time point (i) indicates lighting condition at time of testing (white=light; black=dark). Statistically significant *p*-values ($\alpha \leq 0.05$) are in bold.

(i) Time point	(j) Time point	Mean Difference (i-j)	Standard Error	*p*-Value
**08:00**	12:00	-1.31	7.17	1.000
	16:00	-26.44	7.17	**.029**
	20:00	-53.30	7.17	**.000**
	00:00	-43.33	7.17	**.001**
	04:00	-2.83	7.17	.998
**12:00**	08:00	1.31	7.17	1.000
	16:00	-25.13	7.17	**.039**
	20:00	-51.99	7.17	**.000**
	00:00	-42.02	7.17	**.001**
	04:00	-1.53	7.17	1.000
**16:00**	08:00	26.44	7.17	**.029**
	12:00	25.13	7.17	**.039**
	20:00	-26.86	7.17	**.026**
	00:00	-16.89	7.17	.246
	04:00	23.60	7.17	.056
**20:00**	08:00	53.30	7.17	**.000**
	12:00	51.99	7.17	**.000**
	16:00	26.86	7.17	**.026**
	00:00	9.97	7.17	.732
	04:00	50.47	7.17	**.000**
**00:00**	08:00	43.33	7.17	**.001**
	12:00	42.02	7.17	**.001**
	16:00	16.89	7.17	.246
	20:00	-9.97	7.17	.732
	04:00	40.50	7.17	**.001**
**04:00**	08:00	2.83	7.17	.998
	12:00	1.53	7.17	1.000
	16:00	-23.60	7.17	.056
	20:00	-50.47	7.17	**.000**
	00:00	-40.50	7.17	**.001**

Table S7. Summary of Kruskal-Wallis post-hoc pairwise test on cone myoid length (μm) in light (or subjective light) hours in four lighting regimes. Statistically significant *p*-values ($\alpha \leq 0.05$) are in bold.

(i) Treatment	(j) Treatment	Test Statistic	Standard Error	*p*-Value
**LD**	DD	-14.78	4.97	**.018**
	AdvL	3.89	4.97	.434
	LL	5.78	4.97	.245
**DD**	LD	-14.78	4.97	**.018**
	AdvL	18.67	4.97	**.001**
	LL	20.56	4.97	**.000**
**AdvL**	LD	3.89	4.97	.434
	DD	18.67	4.97	**.001**
	LL	1.89	4.97	.704
**LL**	LD	5.78	4.97	.245
	DD	20.56	4.97	**.000**
	AdvL	1.89	4.97	.704
